# Implications for Tumor Microenvironment and Epithelial Crosstalk in the Management of Gastrointestinal Cancers

**DOI:** 10.1155/2019/4835318

**Published:** 2019-10-31

**Authors:** Yang Ge, C. Benedikt Westphalen, Wen Wee Ma, Kenneth J. Vega, Nathaniel Weygant

**Affiliations:** ^1^Dept of Oncology, Beijing Chao-Yang Hospital, Capital Medical Univ., Beijing, China; ^2^Dept of Oncology, Ludwig Maximillian University of Munich, Munich, Germany; ^3^Dept of Oncology, Mayo Clinic, Minneapolis, MN, USA; ^4^Dept of Gastroenterology, Augusta University, Augusta, GA, USA; ^5^Academy of Integrative Medicine, Fujian University of Traditional Chinese Medicine, Fuzhou, China; ^6^Fujian Key Laboratory of Integrative Medicine in Geriatrics, Fujian University of Traditional Chinese Medicine, Fuzhou, China

## Abstract

Rapid advances in technology are revealing previously unknown organization, cooperation, and limitations within the population of nontumor cells surrounding the tumor epithelium known as the tumor microenvironment (TME). Nowhere are these findings more pertinent than in the gastrointestinal (GI) tract where exquisite cell specialization supports a complex microenvironmental niche characterized by rapid stemness-associated cell turnover, pathogen sensing, epithelial orchestration of immune signaling, and other facets that maintain the complex balance between homeostasis, inflammation, and disease. Here, we summarize and discuss select emerging concepts in the precancerous microenvironment, TME, and tumor epithelial-TME crosstalk as well as their implications for the management of GI cancers.

## 1. Introduction

### 1.1. Gastrointestinal Microenvironment

The gastrointestinal (GI) tract is frequently challenged with exposure to bacteria, parasites, viruses, and other pathogens. For tissue to thrive in these chaotic conditions, it is essential to maintain homeostasis in support of pathogen clearance, digestion, absorption, and efficient cell turnover [1, 2]. This necessity has led to unique tissue compartments with specialized cell types in charge of functions that impact both the GI tract and distant organs including the lung, brain, and others [3, 4]. Imbalances in these compartments as well as deleterious hereditary molecular alterations (e.g., loss of the APC tumor suppressor) can lead to inflammatory, precancerous, and cancerous conditions, and an improved understanding of the factors at play may yield new therapeutic strategies against sporadic and inflammation-associated GI cancers [5].

### 1.2. Inflammation and Injury as a Source of Microenvironmental Instability

Although sporadic and heritable molecular alterations have long been known to be major causes of GI tumorigenesis, recent findings have firmly established inflammation as a hallmark of cancer [6]. Nowhere is inflammatory injury more strongly linked to the development of cancer than within the GI tract where it is implicated in esophageal, gastric, pancreatic, hepatic, intestinal, and other GI cancers. Examples of pathogenic sources of inflammation in these organs include *Helicobacter pylori*, helminths, hepatitis B/C viruses, and various bacterial strains which are able to overpopulate the microbiotic environment under certain conditions [7–10]. Lifestyle factors including smoking, alcohol consumption, processed and red meat consumption, and obesity are also major sources of inflammation which may lead to the expansion of injurious microbiota [11–14].

**Figure 1:**
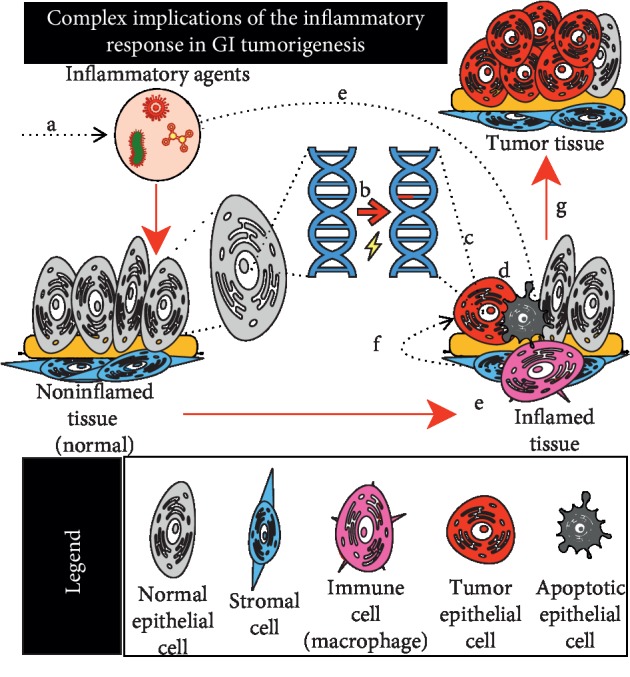
Inflammation and tissue restitution have complex implications for the gastrointestinal microenvironment. (a) The GI epithelium is exposed to a variety of inflammatory agents including bacteria, viruses, parasites, chemicals, and other components which promote injurious shifts in microbial populations and/or directly and (b) induce reactive oxygen and nitrogen species leading to epithelial DNA damage and mutations. Following DNA damage, (c) certain cell types escape DNA repair mechanisms, maintaining these somatic mutations (red), while (d) other cells with effectual DNA repair mechanisms, undergo apoptosis. (e) Macrophages (pink) recruited to the site of injury can engulf pathogens, as well as apoptotic epithelial cell bodies destroyed during inflammation. Macrophage-based detection of signal combinations indicating successful clearance of pathogens (e.g., IL-4 + apoptotic phosphatidyl-serine functional group) can induce (f) macrophage polarization and alternative activation and subsequent anti-inflammatory signaling. (g) Exposure to this signaling may promote tumorigenesis and/or progression in susceptible epithelial cells harboring mutations C or other sources, leading to establishment of tumors and/or metastatic transformation.

A number of inflammatory conditions of the GI tract are thought to prime the tissue microenvironment to give rise to tumors. These include gastroesophageal reflux disease, esophagitis, ulcerative colitis, Crohn's disease, gastritis, pancreatitis, hepatitis, nonalcoholic fatty liver disease, and primary biliary cirrhosis. The protumorigenic activity associated with these diseases is likely mediated by their impact on the DNA damage response, immune signaling, and other mechanisms which may be especially enhanced when these conditions are chronic. Indeed, the inflammatory process itself can be thought of as a double-edged sword in terms of cancer because, whereas inflammation is a source of DNA damage which may support tumorigenesis [15], attenuation of inflammatory signaling may support tumor progression as seen in the alternative activation of macrophages [16] (Figure 1).

### 1.3. Clinical Relevance of GI Inflammation on the Transition to Precancer

Following sustained inflammatory injury in the presence of genetic alterations (e.g., APC mutation), precancerous conditions are often able to take hold in the tissue niche [17]. Examples of this include Barrett's esophagus, intestinal adenomas, pancreatic intraepithelial neoplasia (PanIN), intraductal papillary mucinous neoplasia (IPMN), and others [17–21]. Although inflammatory conditions may progress to precancer which ultimately gives rise to cancer, current knowledge suggests that the majority of patients with acute or chronic inflammatory conditions will not experience progression to cancer. As a result, without additional information (e.g., family history of cancer), knowledge of these conditions is often of minimal practical value in cancer diagnosis, prevention, or prognosis. Moreover, even with consistent endoscopic surveillance in patients deemed to be at high risk, cancer may go unnoticed as seen in colitis-associated colorectal cancer (CRC) [17]. Consequently, preventative strategies including enhanced monitoring, biomarkers, and prophylactic drug therapies have become increasingly desirable. In order to effectively develop these strategies, improved knowledge of precancer and TME cell specialization, microbiotic characteristics, intracellular and intercellular signaling, and other characteristics is needed.

## 2. Targeting Cell Specialization within the Tumor Microenvironment

**Figure 2:**
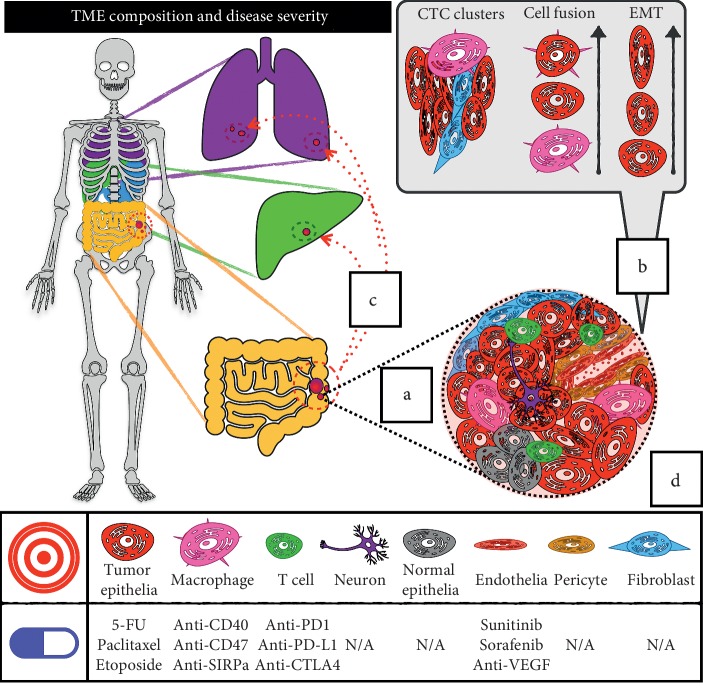
Metastasis and therapeutic efficacy are dictated by complexity within the tumor microenvironment. (a) The TME is comprised of an interacting landscape of unique cell types including tumor epithelia, tumor-associated macrophages, infiltrating T-cells, endothelial cells and pericytes, neurons, and cancer-associated fibroblasts. (b) In metastatic transformation, the TME programs tumor epithelia via EMT, cell fusion, and other processes leading to local invasion and dissemination to distant sites via CTCs and/or CTC clusters. (c) Metastatic dissemination is nonrandom, as in colon cancer where liver metastases are common, although the rules governing this remain to be fully understood. (d) Clinical and developmental drug therapies target or show preference for specific TME components, which factors into their efficacy in various tumor subtypes and combination therapy.

When viewed as an organ, the tumor can be divided into four major cellular compartments: epithelial, stromal, endothelial, and immune. Among these compartments, the tumor epithelium represents the classical “tumor cell,” whereas the stroma, endothelium, and immune compartment comprise the tumor microenvironment (TME). Each of these compartments hosts a variety of cell types with varying functions. The development of single-cell RNA sequencing (scRNA-Seq) technology in recent years has vastly improved the ability to characterize these cells, and their role in cancer initiation and progression is becoming even more apparent [22]. Cancer therapies often target specific cell classes in the tumor and TME (Figure 2). However, due to an early focus on tumor epithelia, there has been limited development of TME-targeted agents. The development of effective immunotherapies (e.g., nivolumab and pembrolizumab) demonstrates the potential for expanding the focus to new TME components. Although this approach has not yet yielded extensive benefit in GI malignancies compared to some other cancers, with further knowledge, these advances will expand the therapeutic arsenal.

### 2.1. Epithelial Cell Types

The majority of cancer research throughout history has focused primarily on the epithelial cell types of the tumor. These cells are often highly proliferative, resistant to apoptosis, and capable of rapidly adapting to insult (e.g., chemotherapy). A variety of studies have shown that tumor epithelial cells are heterogeneous, often containing differing mutations, gene and protein expression profiles, and pathway alterations [23]. Evidence supporting various explanations for this heterogeneity has mounted over the years, and its functional importance in resistance and metastasis is evident. Ironically, despite intense basic and clinical research focused on understanding tumor epithelia, research approaches employing relatively homogeneous populations through the use of commercial cell lines, xenografts, and engineered mouse models have led to a situation in which they are some of the most poorly subtyped cells residing within the TME, and a notable lack of markers to identify various subgroups persists.

Radiotherapy and most chemotherapies were developed to target rapidly proliferating tumor epithelia. Examples of these chemotherapies include taxanes (e.g., paclitaxel and docetaxel), nucleoside analogs (e.g., 5-fluorouracil and gemcitabine), platin drugs (e.g., oxaliplatin and cisplatin), and topoisomerase inhibitors (e.g., irinotecan and etoposide). Typically, these therapies demonstrate an ability to interfere in cell replication through one of the three primary mechanisms: induction of DNA damage, interference in microtubule dynamics, or inhibition of DNA synthesis. Many of these antiproliferative therapies have been approved in GI cancers. However, due to adaptive mechanisms within the tumor as well as toxicity to normal cells, they often provide limited benefit. Tumor epithelia may adapt to treatment via quiescence or slower cell division, or through the expression of drug efflux machinery [24]. These characteristics are often linked to epithelial-mesenchymal transition (EMT) and cancer stem cells (CSCs), which are capable of recapitulating the tumor with increased metastatic capabilities and drug resistance. Despite these shortcomings, these compounds may increase patient survival, and over the past decade, it has become clear that combinations of these drugs may be more effective than individual agents. This is especially notable in pancreatic cancer where recent studies show that the combination of 5-fluorouracil, oxaliplatin, irinotecan, and leucovorin (FOLFIRINOX) can dramatically improve pancreatic ductal adenocarcinoma (PDAC) survival [25, 26].

### 2.2. Stromal and Endothelial Cell Types

Stromal cells provide structure to an organ, and the tumor is no exception. Subtypes with known functional importance in the TME include fibroblasts and pericytes. Cancer-associated fibroblasts (CAFs) surround tumor epithelia providing physical structure, secreting extracellular matrix (ECM), and directing various tumor processes. Additional fibroblasts can be continually recruited from tumor stroma and normal tissue, and CAFs are a primary building block for desmoplasia, which compromises the delivery of conventional and targeted therapies to the tumor [27]. In GI cancers, CAFs are implicated in molecular regulatory processes including cytokine/chemokine secretion, immune checkpoint, tumor growth factor signaling, macrophage polarization, and angiogenesis [28].

Aside from CAFs, mesenchymal pericytes also provide structure within the tumor by maintaining a skeleton for endothelial vessel formation. Interestingly, some findings support the possibility of epithelial-pericyte transition occurring within the TME to support tumor processes such as angiogenesis [29]. Overall, research into cancer-associated pericytes remains limited, but given their role in normal tissue and noncancer diseases, and the importance of angiogenesis in tumorigenesis and progression, a better understanding will likely improve TME targeting strategies [29, 30].

Endothelial cells form blood vessels supported by pericytes and are a key transit point for migrating cells and signaling factors entering and exiting the tumor. Their prevalence is associated with poor outcomes in many cancer types including GI cancers, and high levels of vascularization such as found in clear cell renal carcinoma (ccRCC) and PDAC are associated with potent resistance to chemotherapies [31]. The prominent VEGF pathway is perhaps the best known target within this system [32], but our understanding of tumor-associated endothelia remains limited.

Endothelial cells along with the VEGF, PDGF, and several linked pathways are key components involved in the process of angiogenesis. Angiogenesis pathways interact via a variety of ligand-receptor interactions and are activated by hypoxia-inducible factors. Canonically, hypoxic TMEs induce the expression of transcription factors HIF1A and HIF2A (EPAS1), which in turn upregulate VEGF expression. VEGFs bind to VEGF receptors which regulate endothelial cell viability and migration, recruit immune cells to the tumor, and support lymphangiogenesis [33]. Functionally, angiogenesis within the tumor was recognized early on as a contributor to disease progression and mortality, and vascularization offers additional routes for nutrient uptake supporting growth, as well as dissemination of circulating tumor cells (CTCs). Among GI cancers, this process is especially important in HCC [34], as well as in PDAC where it supports desmoplasia [35], preventing drugs from easily accessing the TME [35]. Based on the importance of this mechanism, a class of angiogenesis inhibitors was developed to target VEGF, PDGF, and related signaling pathways. The most prominent of these inhibitors, sunitinib, has found use in a variety of tumors including PDAC [36]. Other commonly used antiangiogenesis agents include sorafenib, regorafenib, aflibercept, and the anti-VEGF monoclonal antibody (mAB), bevacizumab, which are indicated in some CRCs [33].

### 2.3. Infiltrating and Auxiliary Immune Cells

Perhaps the most prominent emerging topic in TME research is the immune system. Immune cell subtypes present in tumors include T cells, B cells, natural killer (NK) cells, macrophages, and myeloid-derived suppressor cells (MDSCs) among others. The presence and activation status of these components are key controllers of tumor fate and the response to therapies. A variety of immune subset-specific processes act as levers in this system, including alternative activation of macrophages, presentation of antigens by major histocompatibility complex (MHC), PD1/PD-L1 interaction, and others [37]. Moreover, dated knowledge of immune cell types is slowly being expanded and clarified by scRNA-Seq and other profiling studies demonstrating unique subpopulations of each and expanded hematopoietic and tissue-resident differentiation pathways.

Novel therapies targeting immune-tumor interactions are emerging as the treatment of choice in a variety of cancers. Currently, immunotherapies are more commonly used after conventional first-line therapy but are expected to supplant some of these in the future. Despite the availability of target specific and well-tolerated immunotherapies, and the known importance of the immune system in the GI TME, they have thus far fallen flat relative to the expectations set in clinical trials of some non-GI cancers. However, there are use cases that demonstrate their potential, and effective companion biomarkers and an expanded understanding of the complexities of immune-GI TME interactions may overcome existing challenges to this approach.

Microsatellite instability (MSI) and mismatch repair deficiency (MMRd) may define some immunotherapy-susceptible subsets of GI cancer patients. In MMRd CRCs, PD1-targeted pembrolizumab contributes to significant improvements in progression-free survival through an apparent immune mechanism [38]. In PDAC, similar studies targeting immune checkpoint have demonstrated limited proof of concept for anti-PD1 combination therapies in some patients [39]. Analyses also suggest that advanced gastric cancers may be susceptible to anti-PD1 mAB therapy, which can increase overall survival and reduce adverse events [40]. Taken together, these findings demonstrate the potential for immunotherapies and specifically immune checkpoint inhibitors in the treatment of GI cancer. However, advancing the use of immunotherapies in GI cancer will require combination with chemotherapy, as well as identification of susceptible tumors using specific biomarkers (e.g., PD-1 positive/negative, MSI-H/L, and high/low tumor mutational burdens) and from among the defined tumor subtypes that describe the origin and TME context of disease [41, 42].

## 3. Cell Interactions, Transformation, and Displacement within the TME

The TME and its associated tumor epithelium is a dynamic compartment driven by intratumoral and extratumoral signaling regulating metabolism, secretory functions, cell populations, and ultimately progression. Exploiting the TME for improved therapies and monitoring will require an understanding of these specific processes and any signaling crosstalk that may occur between other TME components and tumor epithelia. Cell communication occurs through a variety of autocrine, paracrine, and juxtracrine signaling mechanisms, and signals may be transmitted across long distances (e.g., between discrete organs). Examples of signaling molecules with great import within the TME include cytokines and chemokines, growth factors, and immune-related ligands. Methods for conveying these signals include extracellular vesicles, traditional secretion, and membranous ligand expression. Select signaling pathways and processes of great importance within the GI TME include EMT, CSC-mediated signaling, the formation and dissemination of CTCs, immune-modulatory activities, and potentially organ-organ biological axes.

### 3.1. Epithelial-Mesenchymal Transition and CSCs within the TME

First identified as an embryonic developmental mechanism, EMT is a process by which tumor epithelia can mimic essential TME cellular components and is now widely considered one of the most important pathways in GI tumor progression. During EMT, stimulation of various signaling pathways leads to the expression of a set of transcription factors (ZEB1/2, SNAI1/2, and TWIST1/2) which remodel tumor epithelial cells to transitional and then mesenchymal cell types characterized by loss of tight junction proteins and expression of mesenchymal markers (e.g., vimentin and fibronectin) [43]. Mesenchymal cells have significantly greater migratory capacity compared to epithelial cells, and evidence suggests EMT is a primary driver of invasion and dissemination leading to metastasis. However, it is less clear how circulating tumor cells (CTCs) that have undergone EMT are able to initiate mesenchymal-epithelial transition (MET) and effectively colonize the distant metastatic site [43]. Moreover, EMT is dynamic within the TME, and transitions can occur in both directions (EMT or MET) in response to environmental stimuli. For example, epithelial cells are often less resistant to chemotherapy agents and EMT may allow them to adapt and escape apoptosis through ABC drug efflux transporter expression and a more mesenchymal-like phenotype. Finally, EMT is inextricably linked to stemness and CSCs, and activation of EMT may potentially induce dedifferentiation of tumor epithelia [43].

Though the existence of CSCs was long hypothesized, the stochastic model of tumorigenesis and progression championed by the Vogelstein group was favored until work by the John Dick lab demonstrated the existence of CSCs definitively by identifying a specific AML subpopulation (CD34^+^/CD38^−^) giving rise to the totality of AML cell types [44]. Like normal stem cells, CSCs are defined by self-renewal and pluripotency and within the TME, they can control tumor progression not only through signaling but also by regulating the overall composition of tumor epithelia through their progenitors. Perhaps the most well-described CSCs in the GI tract are the intestinal crypt base normal stem cells (NSCs) [45] and the terminally differentiated (in normal tissue) intestinal tuft cell [46]. Both of these populations can initiate tumors (cell of origin) in the presence of mutation (e.g., APC loss), while current research suggests that the tuft cell-derived CSC may be more specific to inflammation-associated cancer [47]. However, since tuft cells arise from NSCs, it is probable that these phenomena are inextricably linked and they may need to be studied in tandem. Importantly, the activity of both of these cell types in inflammatory, precancerous, and cancerous conditions may provide an opportunity to understand how different CSC populations interact with immune and other components in the tumor and surrounding tissue.

### 3.2. CTCs, Dissemination, and Metastatic Colonization and Progression

The importance of the TME to metastatic colonization and progression was first hypothesized in the late 19^th^ century when Stephen Paget proposed what became known as the “seed and soil hypothesis.” Through studying many hundreds of cancer patient autopsies, Paget determined that metastasis does not occur by chance. Instead, certain organs provide an optimal environment for this form of cancer progression [48]. Recent findings suggest that the primary tumor may prepare these sites for metastasis at a distance via endocrine signaling, but it must also effectively transform and disseminate cells to these distant sites. Both CSCs and EMT are known to be major factors in transitioning primary tumor cells to CTCs [49], but previous research into CTC biology leaves much to be desired.

Most CTC studies have depended on the use of FACS sorting to identify and characterize these cells. However, FACS techniques have traditionally depended on dissociation to single cells. New findings are demonstrating that CTCs benefit from traveling through the bloodstream in clusters with other CTCs and supportive TME cells including immune cells, fibroblasts, and endothelia [50–52]. However, these CTCs were previously envisioned as a kind of spore for metastasis; these findings suggest that CTC clusters may be more comparable to a metastatic ship containing components and provisions to support the journey from one continent (primary tumor) to another (distant site). Emerging findings also suggest the novel possibility that tumor cell fusion (e.g., macrophage-epithelial fusion) and/or tumor epithelial mimicking of specialized cell functions are involved in the preparation and transport of CTCs to distant sites [53, 54]. However, many pressing questions remain regarding the clonal nature of CTCs and their progeny, the influence of paracrine and endocrine signaling on CTC processes, and the physical properties of CTCs.

### 3.3. Local and Discrete Immunomodulation in the TME

Infiltrating and auxiliary immune cells regulate cancer through a variety of mechanisms. The most prominent example of this currently is the PD-1/PD-L1 interaction which tumor epithelia and other cells within the TME exploit to activate apoptosis in cytotoxic T cells programmed to destroy them. Modulation of the immune system can be initiated from many different TME components. Secretory products such as cytokines and chemokines are often key regulators of these processes, and cascading events complicate knowledge of the overall picture. Studies of the inflammatory and precancerous intestinal microenvironment may be one of the best systems to consider when pondering these complex cell and molecular network interactions because of the intestinal epithelium's well-described cellular structure and function.

The intestinal epithelium contains six well-documented cell types: stem cells, antibiotic-producing Paneth cells, hormone-producing enteroendocrine cells, mucin-producing goblet cells, absorptive enterocytes, and sensory tuft cells. Recent studies also provide evidence for functional subgroups of stem cells [55] and tuft cells [56] and an injury-specific reserve stem cell [57]. When challenged by injury, tuft cells sense epithelial damage and respond by secreting IL-25. This IL-25 is detected by innate lymphoid type II cells (ILC2s) through IL-25 receptor (IL17-Rb), which in turn secrete IL-4/13. These secreted interleukins interact with IL-4 receptor on intestinal epithelial stem cells, reprogramming them to produce sensory tuft cells and goblet cells which secrete mucins to protect the intestinal epithelial barrier [58]. As a whole, this system functions in a self-contained fashion to protect and repair the intestinal epithelium, but as a consequence tuft cell hyperplasia occurs, likely increasing the potential for CSC transformation. Given the long-lived nature of some tuft cells [47], this risk may continue long after inflammation subsides, and as tuft cells are also present in GI tumors, this mechanism may be a significant factor in epithelial-immune crosstalk within the TME.

### 3.4. Evasion of Antitumor Immune Mechanisms within the TME

Several mechanisms allow tumor epithelial cells within the TME to escape detection and eradication by the immune system. Immune cells depend on the presentation of antigens to detect, home to, and neutralize an aberrant cell within the tissue. This process can be subverted by inhibiting antigen presentation machinery directly on tumor epithelial cells [59], deactivating immune antigen-presenting cells (such as dendritic cells), intercepting cytotoxic CD8^+^ T-cells and natural killer cells, avoiding autophagy from macrophages, activating CAF-based desmoplasia, and other mechanisms [60]. Moreover, the composition of immune cells within the TME is a key facet in tumor progression and response to therapy. This includes patterns of cytotoxic T-cell tumor infiltration, recruitment of immunosuppressive myeloid-derived suppressor cells (MDSCs) and *T* regulatory cells (Tregs), and reprogramming of macrophages towards an anti-inflammatory phenotype [60].

Despite a wide variety of innate and adaptive mechanisms by which the immune system maintains surveillance for cancer, tumors are notoriously successful at avoiding immune-based detection. Without expression of major histocompatibility complex (MHC) class I or with damage to associated antigen peptide transport, the immune system's ability to detect aberrant cells, including tumor cells, is highly limited [59, 60]. Essential components in this process include endoplasmic reticulum-based chaperones calnexin and tapasin. These chaperones assist in the transport of peptides and preparation of the trimeric complex of B2-microglobulin, MHC class I heavy chain, and antigen peptide. When presented on the surface of the cell, T-cell receptors (TCRs) detect this complex and perform their associated tasks, and its expression is often associated with improved responses to chemotherapies and immunotherapies, while loss of expression or alterations to antigen presentation machinery can result in resistance to therapy [59].

Various signaling pathways are involved in regulating the tumor immune response and cancer immunosurveillance [60]. Many of these pathways are common to both immune and other TME cells as well as tumor epithelia. Therefore, attempts to target them must take their activity in multiple cellular compartments into consideration. Examples of multicompartment pathways involved in regulating cancer immunosurveillance include MAPK, WNT, PI3K, and STAT3 signaling pathways [60]. For example, IL-6-mediated STAT3 activation is a key driver of M1 (proinflammatory) to M2 (anti-inflammatory) macrophage transition [61] and an active tumor epithelial pathway that directs proliferation and metastasis [62]. Tumor secretory factors driven by molecular signaling pathways often directly regulate TME components. Prominent examples regulating the immune compartment include TGFB, PGE2, and VEGF [60]. Variability in these pathways is one of the reasons that advanced molecular subtyping and personalized therapy is likely to hold great potential in GI cancers.

Individual TME cell types and structural components have varied roles in manipulating cancer immunosurveillance. Mounting evidence suggests that CAFs are able to recruit M2-like macrophages, MDSCs, and Tregs and remodel the TME towards an immunosuppressive and protumorigenic phenotype [63]. Moreover, they may express immune checkpoint markers PD-L1/2 and directly interfere in natural killer cell-mediated cytotoxicity and are in part responsible for defining and altering the properties of extracellular matrix (ECM) [63, 64]. Aside from structural fibers that support the tumor in three-dimensional space, ECM holds a mixture of growth factors, enzymes, and signaling molecules [64, 65] which can regulate the activity of tumor-associated macrophages and entice colonization of the tumor site by endothelial and immune cell subsets [63, 64]. Importantly, it also functions as a track for the migration and invasion of tumor epithelial cells that have undergone EMT as they attempt to disseminate to distant sites [64]. Taken together these concepts demonstrate the importance of the TME to evasion of cancer immunosurveillance.

### 3.5. TME and Organ-Organ Axes

Recently, functional organ-organ axes (e.g., gut-lung axis and gut-brain axis) have been described in nontumorigenic contexts [3, 4, 66–68], but this concept remains controversial in cancer. Surprising findings in this field demonstrate the importance of the gut microenvironment to cognition and neurological disorders (autism, addiction, and depression) [3, 66], nongut inflammatory conditions (asthma and COPD) [69], and liver diseases (e.g., cirrhosis) [67] among others. The limited research findings in this area tend to focus more directly on microbiotic populations, but the implications for the GI TME merit interrogation of the contribution of mammalian cells and pathways to these axes and their drivers.

Signaling between the gut and distant organs is hypothesized to occur through pathogenic and/or immune mechanisms. In the pathogenic mechanism, alterations in populations of gut bacteria caused by stimuli such as altered diet, increased stress, or gut disorders can result in secretion of bacterial products and microenvironmental remodeling characterized by altered pH, increased barrier permeability, and the exposure of organs outside of the gut to the by-products of these alterations [68]. The immune component is thought to be directed by pathogens and, by proxy, the response to their presence occurring through secretion and sensing, priming of progenitor populations for differentiation, and activation of mature populations leading to or orchestrated by molecular and functional alterations in immune cells [70].

Extrapolation of findings concerning inflammatory innate lymphoid type II cells (iILC2s) makes a case for considering the impact of gut-organ axes in the tumor and metastatic microenvironment. Until recently, ILC2s were thought of as resident within each tissue and suspected to originate in the bone marrow. However, iILC2s which arise in the presence of IL-25 or IL-25 stimulating pathogens do not exist in appreciable numbers at homeostasis but become plentiful upon IL-25 stimulation in multiple organs including the gut, lung, and liver. Recent findings trace the origin of these cells to the gut lamina propria, where they are enticed to migrate to distant organs by lipid-mediated chemotaxis [71]. These findings imply that inflammation within the gut can have significant consequences not only to the GI tract and TME but also to non-GI TMEs. Interestingly, activation of ILC2s, which is necessary to protect the gut epithelium during injury, not only regulates the fate of epithelial progeny by stimulating stem cells [58] but also maintains macrophage-dependent immunity [72]. Both of these processes may play an important role in tumor progression and drug resistance, and preclinical evidence indicates that blocking IL-25 signaling through its receptor (IL-17Rb) using a mAB may be an effective therapeutic route in PDAC [73]. However, in another study, direct neutralization of IL-25 with a mAB in a chemically induced inflammatory model of CRC supports tumor progression [74], suggesting that the response to IL-25 therapy would likely be heavily dependent on the characteristics of the patient's TME.

## 4. Future Directions for GI TME Research and Development

In the immediate term, pressing obstacles to overcoming GI TME protumor mechanisms in the clinic include testing the expanded use of drug combinations like FOLFIRINOX, devising methods to identify patient subsets that may benefit from immunotherapy, and improving management and therapy through precise classification of tumors, development of biomarkers, and integration of computational technologies into clinical workflows. Beyond the immediate term and *in silico* advances such as scRNA-Seq, deep-learning, and advanced imaging technologies, the development of new therapies with increased specificity against traditional biochemical targets, engineered biological therapies like CAR-T and mABs, and others are expected to expand survivability and improve outcomes. Overall, a more holistic understanding of molecular, microenvironmental, environmental, and behavioral contributors to inflammatory damage and cancer in the GI tract is needed to expand translational and clinical applications and prevent and/or delay tumorigenesis and progression.

### 4.1. Emerging Clinical Importance of the TME

The TME is perhaps the most difficult component for clinicians to monitor in GI cancers. Noninvasive imaging techniques are currently useful to assess macroscopic changes at the organ level but not yet sufficient to identify changes in the TME in most cases. The ability to analyze the TME directly is also compromised by static access to tissue, which can often only be obtained at predetermined times such as following diagnostic biopsy and surgical intervention. Moreover, the TME is not homogeneous, so information gleaned from these specimens may be of limited clinical value. As a result, it will be necessary to develop therapies that target specific TME components and biomarkers that provide a surrogate measure to monitor changes during the course of therapy.

The majority of novel drug classes currently being developed to target the TME falls under the category of immunotherapies. As discussed in the previous paragraph, immune checkpoint inhibitors have shown success in limited subsets of GI tumors, especially CRCs demonstrating MSI or defective mismatch repair [38–40]. High MSI (MSI-H) is characterized by hypermutation linked to MLH1 promoter hypermethylation but can also be induced by hereditary mutations to mismatch repair machinery [75]. Perhaps counterintuitively, the increase in mutations results in enriched neoantigen presentation, making the use of immune checkpoint therapies possible as seen in MSI-H CRC. These therapies work by neutralizing CTLA4 or through blocking the interaction between PD-L1 (CD274), expressed on tumor epithelia and surrounding supportive cells, and PD-1, expressed on infiltrating CD8^+^ cytotoxic T-cells, a process that is regulated by MHC class I and the components of the neoantigen presentation apparatus. Indeed, ongoing clinical trials are demonstrating promise for these immunotherapeutic agents with or without combination therapy, and the success of PD-1/PD-L1 axis therapies has invigorated the pursuit of targets exploiting other immune-tumor interactions [76]. Prominent emerging targets in this field of development include CD47 [77], CD40 [78, 79], interleukins such as IL-10 and IL-17 [73, 80], inflammatory mediator IDO1 [81], and a variety of engineered viral, vaccine, and cell therapies.

Currently, clinical trials are underway to investigate macrophage targets CD47 and CD40 in GI cancer. CD47 is a highly expressed tumor epithelial extracellular ligand that is detected by the macrophage SIRP*α* receptor. This “do not eat me” signal activates phosphatases SHP-1/2 resulting in inhibition of autophagy and ultimately protection of the tumor epithelial cell. CD47 or SIRP*α* mABs can bind and prevent this interaction, allowing effective autophagy [77]. CD40 expressed on tumor cells can be stimulated by agonist CD40L (CD156) to directly induce apoptotic cell death. In the immune compartment, CD40 activation on dendritic cells leads to the recruitment of tumor-targeting cytotoxic T-cells, and on B-cells, it stimulates endogenous antitumor mAB production. Similarly, in macrophages, CD40 activation leads to cytokine and chemokine secretion, which may have antitumor activity in some contexts. Both CD40 agonist mABs and ligands can be used to simulate this activity [78, 79]. Together, CD40 and CD47 are prime examples of clinical development targets leveraging TME knowledge gleaned from decades of basic laboratory research and are an important step towards phagocytosis modulating anticancer drugs.

Secretory products from the TME are also major drug development targets in oncology. Among these, interleukins are perhaps receiving the most attention at the moment, and evidence for targeting chemokines and their receptors is accumulating. Pegylated IL-10 is one such example (pegilodecakin; AMA0010) that is currently being tested. In PDAC clinical trials, pegilodecakin is able to increase the activation of cytotoxic T cells and improve overall survival when combined with FOLFOX [82]. Further assessments of interleukins in cancer therapy are largely occurring at the preclinical level, with promising findings for targets such as IL-25 [73, 80] as previously discussed. Fortunately, the prior interest in these and related targets for psoriasis, asthma, and inflammation-related disorders has resulted in a variety of existing therapies [83], including mABs with known human safety profiles that could be repurposed for cancer immunotherapy.

Finally, more traditional intracellular biochemical targets with the ability to regulate TME components are being studied. IDO1 is a key enzyme in the conversion of tryptophan to kynurenine. When levels of IDO1 enzyme are high in the TME, cytotoxic T cell and NK cell functions are suppressed, regulatory T cells are activated, and MDSCs expand. Inhibiting the activity of the enzyme can reverse this process and enhance antitumor immunity [81, 84]. Various compounds in this class are being used in clinical trials in liver, pancreatic, and other tumors. Similar to PD1-targeted immunotherapies, IDO1 agents are expected to have therapeutic potential in subsets across the solid tumor spectrum, and companion biomarkers and identification of susceptible tumor subtypes will likely be essential to IDO1-targeted therapy. Additionally, given the mechanism of action, IDO1 inhibitors are also being trialed in combination with immunotherapies such as nivolumab and pembrolizumab [84].

### 4.2. TME Biomarker Concepts and Personalized Medicine

The effective development of predictive biomarkers utilizing next-generation technologies has only recently become possible. The clinical potential for these markers is demonstrated by the use of the Prosigna (formerly PAM50), Oncotype Dx, and Mammaprint tests in hormone-positive breast cancers. Moreover, the development of comparable GI cancer markers is progressing rapidly as evidenced by the predictive abilities of the consensus molecular subtypes (CMS) developed by the CRC subtyping consortium. These 4 subtypes are characterized by MSI and immune activation (CMS1), canonical signaling through WNT (CMS2), dysregulated metabolism (CMS3), and TGFB and stromal/angiogenic characteristics (CMS4) [42]. These findings not only are improving the understanding of the initiation and progression of CRC but also have practical implications for the treatment and monitoring of patients bearing tumors meeting these biomarker criteria. For instance, standardized CMS subtyping routines provided for RNA-Seq data may be applied to clinical trial data to identify tumor subtypes that are susceptible to therapy. This may lead to approval of drugs that were effective in specific subtypes, but not robust to overcome progression in all 4 CMS subtypes [42]. A handful of clinical trials targeting the CRC CMS4 subtype with novel immunotherapies including anti-PD-1 mAB spartalizumab [85], dual PD-1/TGFB engineered mAB-fusion protein M7824 [86], and a dendritic cell vaccine (AVEVAC) [87] have already begun in the US and EU. Similar subtyping advances in pancreatic [41], gastric [22], and liver cancers leveraging data accumulated from large-scale multicenter projects including the Cancer Genome Atlas (TCGA), International Cancer Genome Consortium (ICGC), and others hold similar potential.

Inflammatory biomarkers are another key area of development exploiting the properties of the GI TME. These can be detected by a variety of methods in the tumor tissue, serum, and from other sources. Examples of these include standardized ratios of immune cell types (e.g., neutrophil, lymphocyte, and platelet), levels of circulating cytokines/chemokines, and transcriptome-based subtyping of inflammatory subtypes (e.g., CMS1 CRC (Bailey et al 2016 immunogenic PDAC subtype)) [41, 42, 62, 88, 89]. Gardini et al. identified hepatocellular carcinoma (HCC) patients with increased neutrophil-to-lymphocyte ratio (NLR) or systemic inflammation index (platelet count × NLR) as prone to disease progression when undergoing sorafenib therapy compared to those with lower ratios. Adjusting for other relevant clinical factors demonstrated the independence of this prognostic measure [89]. Similar findings with NLR in metastatic CRC demonstrate its potential to predict objective response to therapy over multiple courses of treatment (1^st^–3^rd^ line) [88]. Apart from direct measurements of immune cell types, secreted cytokines and chemokines are potential surrogates for immune activity in the TME including in response to drugs. For example, elevated serum IL-6 levels are indicative of increased risk of HCC and PDAC as well as predictive of PDAC progression [62]. Overall, exploiting objective measures of TME inflammatory characteristics is likely to further personalize therapy in GI cancers.

Concurrent to advances in expanding the use of inflammatory biomarkers and deciphering the underlying molecular characteristics of GI tumors through accumulation of data, improvements to sequencing technology, machine-learning subdisciplines, and imaging are heralding an era of precision medicine. The use of scRNA-Seq in particular is poised to dimensionally expand knowledge of the GI tumor niche and subtypes and increase the practical value of basic research findings. The two major obstacles to the development and practical use of new and existing next-generation biomarker technologies are limitations in obtaining appropriately sized cohorts with suitable sample quality for analysis and prohibitive financial costs. However, the ability to overcome these obstacles is well within sight, and costs for sequencing are decreasing by the year. Finally, as in previous phases of research and development, these maturing technologies will seed new technologies that will fundamentally shift the approach to biomarkers, such as the recently described Slide-Seq technique which combines standard tissue pathology and scRNA-Seq [90] and other multidimensional techniques.

## 5. Conclusions

GI cancers are increasingly prevalent worldwide, and the importance of the health of the GI tract in these and non-GI cancers is becoming more apparent. Currently, inflammation is reemerging as a focal point, as the role of the immune system and efficacy of new immunotherapies have taken center stage in oncology. Advances in basic research and technological innovations such as scRNA-Seq, which dimensionally expand our understanding of the TME, are beginning to provide a more holistic concept of GI tumorigenesis and progression. The knowledge gleaned from these advances will support a new generation of therapies and diagnostics, which should enable a breakthrough era of personalized GI cancer management and lead to improved quality of life and survival. In the coming years, basic and clinical researchers should focus on leveraging this growing knowledge to more completely uncover the structure and mechanics of the GI TME and to increase the precision of therapeutic intervention for GI cancer patients.
